# Photochemical Rearrangements of Pyridine N-Oxides: Pathways to Oxaziridine Derivatives

**DOI:** 10.3390/molecules30244776

**Published:** 2025-12-14

**Authors:** Cristian J. Guerra, Yeray A. Rodríguez-Núñez, Efraín Polo-Cuadrado, Mitchell Bacho, Jorge Soto-Delgado, Victor B. Fuentes-Guerrero, Eduardo I. Torres-Olguín, Cristopher A. Fica-Cornejo, Daniela Rodríguez-García, Manuel E. Taborda-Martínez, Leandro Ayarde-Henríquez, Adolfo E. Ensuncho

**Affiliations:** 1 Laboratorio de Síntesis y Reactividad de Compuestos Orgánicos, Departamento de Ciencias Químicas, Facultad de Ciencias Exactas, Universidad Andrés Bello, República 275, Santiago 8370146, Chile; 2 Laboratorio de Diseño y Síntesis de Compuestos Bioactivos, Departamento de Química Orgánica, Facultad de Ciencias Químicas, Universidad de Concepción, Concepción 4070386, Chile; 3 Departamento de Ciencias Biológicas y Químicas, Facultad de Medicina y Ciencia, Universidad San Sebastián, Campus los Leones, Lota 2465, Providencia, Santiago 7510602, Chile; 4 Departamento de Ciencias Químicas, Facultad de Ciencias Exactas Bello, Universidad Andrés Bello, Quillota 980, Viña del Mar 2531015, Chile; 5 Facultad de Ciencias de la Educación, Universidad del Magdalena, Carrera 32 No. 22-08, Santa Marta 470004, Colombia; 6 School of Physics, Trinity College Dublin, 2 D02 PN40 Dublin, Ireland; 7 AMBER—Advanced Materials and BioEngineering Research Centre, 2 D02 PN40 Dublin, Ireland; 8 Grupo de Química Computacional, Facultad de Ciencias Básicas, Universidad de Córdoba, Carrera 6 No. 77-305, Montería-Córdoba 230001, Colombia

**Keywords:** thermochemistry, photochemical rearrangements, ab initio calculations, charge redistribution

## Abstract

The photochemical behavior of substituted pyridine N-Oxides is characterized by complex rearrangements culminating in the formation of valuable photoproducts. The CAS(10,8)/cc-pVDZ approach with NEVPT2 corrections is applied to investigate geometric distortions associated with the $S_{1}$ excited state, conical intersections, and the ultimate transformation of pyridine N-Oxides into oxaziridine-like derivative formations. Our results reveal that the deactivation of the $S_{1}$ excited state is driven by an out-of-plane rotation of the N-O oxygen atom, resulting in the formation of a lone pair over the nitrogen atom. Along this excited-state reaction pathway, the N-O bond undergoes significant weakening, while a C=C double bond emerges mainly in the excited state. The deactivation at the minimum-energy conical intersection leading to the ground state reveals the formation of an oxaziridine-like intermediate, which subsequently converts into a 1,2-oxazepine derivative.

## 1. Introduction

**Scheme 1:**
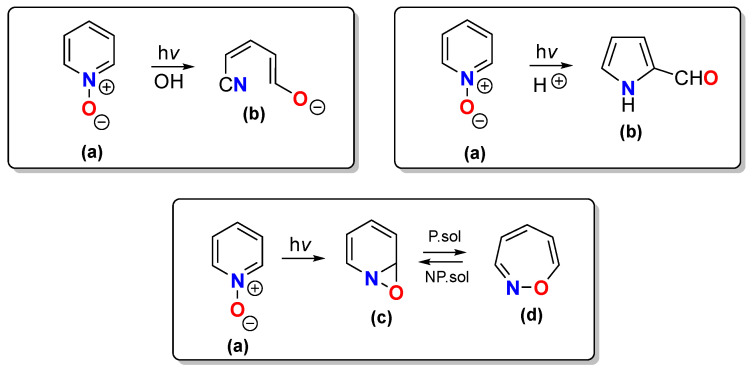
Photoproducts observed upon irradiation of pyridine N-Oxides (**a**) under various reaction conditions: 4-cyanobuta-1,3-dien-1-olate derivatives (**b**), oxaziridine (**c**), and 1,2-oxazepine (**d**).

The photochemistry of the N-Oxide (NOx) function comprises a wide variety of chemical reactions, such as ring expansion [1,2], ring-cleavage [3], oxygen shift [4], and electronic rearrangements [5,6,7]. Pyridine NOx chemistry has recently gained substantial attention owing to its applications in photocatalysis [7,8], selective functionalization [8,9,10], and cross-coupling [11], which offer sustainable and selective approaches in organic synthesis. Photoproducts resulting from the irradiation of pyridine NOx depend on the solvent employed [12,13,14]. For example, the light-induced treatment of pyridine NOx in aqueous hydroxide solutions mainly forms 4-cyanobuta-1,3-dien-1-olate derivatives [13] (b), as shown in Scheme 1. Conversely, conducting this reaction in an acidic medium results in the formation of pyrrolaldehyde, and additional products can be observed when the pyridine ring is modified [14].

The extensive array of photoproducts discussed above highlights the significant complexity involved in elucidating the photochemical transformations of N-Oxides [3,12,15]. For intramolecular rearrangements, these processes are generally thought to occur in the lowest-energy singlet excited state [16]. This state corresponds to an *n* → $\pi^{*}$ electronic transition, characterized by the migration of electron density from the N–O group to the aromatic ring [12,17]. One of the most relevant photochemical reactions that pyridine NOxs experience in organic solvents is ring expansion, yielding 1,2-oxazepine derivatives [18,19]. The production of 1,2-oxazepine derivatives was strongly influenced by the polarity of the solvent, resulting in higher yields in polar solvents. Harran and colleagues observed the formation of oxaziridine by irradiating pyridinophane NOx species. The latter then underwent a ring expansion, leading to 1,2-oxazepine [20]. Recently, this synthetic strategy was employed in 1,2-diazepines production from pyridinium ylide irradiation [21], highlighting its significant relevance.

The formation of 1,2-oxazepine, like other photoproducts, depends on the presence of reaction intermediates, such as oxaziridine-like compounds [3,6,15,18,19,22]. The equilibrium between these species is influenced by external factors. Notably, the type of solvent is critical: a protic solvent favors the formation of 1,2-oxazepine by shifting the equilibrium towards its formation [18,19,23]. The mechanistic implications of 1,2-oxazepine formation remains insufficiently documented in the literature, particularly regarding the stages involving excited states before this process. Both the chemical reaction leading to 1,2-oxazepine and the internal conversion from the excited singlet state proceed with high efficiency [23,24]. In contrast, fluorescence and intersystem crossing to the triplet state are notably inefficient, suggesting significant structural distortion upon excitation [12]. This behavior is consistent with the $n\to \pi^{*}$ or charge transfer (CT) characteristics of the singlet state, resulting in a pronounced loss of aromaticity and, likely, planarity [16].

This study aims to bridge the gap in our understanding of the detailed mechanistic pathway leading to 1,2-oxazepine formation, emphasizing the formation of oxaziridine-like intermediates from the $S_{1}$ excited state, using ab initio methods. The findings offer new insights into the photochemical behavior of pyridine NOxs and expand their potential applications in synthetic photochemistry.

## 2. Results and Discussion

Our theoretical study primarily examines the NOx system, which serves as a platform for mechanistic discussion. The exploration of substituted motifs further supports the discussion and reliability of the results. In the context of NOx photochemistry, the vertical $n\to \pi^{*}$ transition facilitates the population of the $S_{1}$ excited state, where the electronic rearrangement characteristics of these compounds are suggested to occur. Table 1 presents the wavelengths, excitation energies, and oscillator strengths (Focs) calculated at the NEVPT2(10,8)/cc-pVDZ level. This method has been shown to provide reliable physical parameters, including vertical excitation energies in photo-induced chemical reactions [25,26,27].

**Figure 1:**
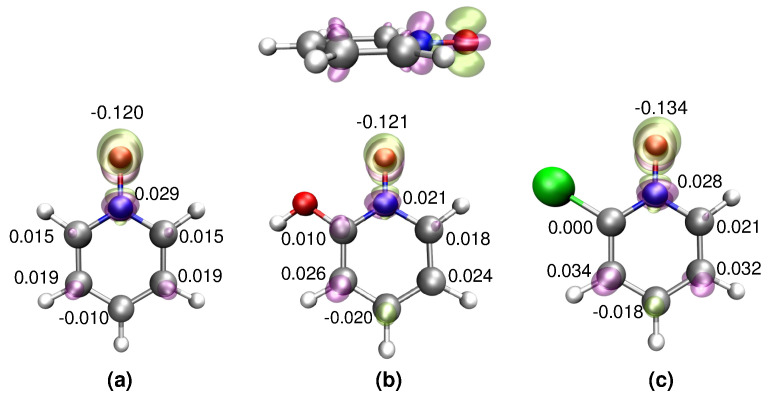
Isosurfaces of the electronic redistribution function $\Delta \rho_{ij}\left(r\right)=0.04$ ($\Delta \rho_{ij}\left(r\right)>0$ in pink, $\Delta \rho_{ij}\left(r\right)<0$ in green) for the NOx (**a**), NOx-o-OH (**b**), and NOx-o-Cl (**c**) systems. The values of $\langle N_{ij}^{k}\rangle$ condensed on the pyridine ring atoms and the N-O group are also shown.

Experimental studies of the photophysical properties of NOxs indicate that the absorption band of interest ($S_{0}\to S_{1}$) lies within the range of 270–330 nm for substituted NOxs [6,10,15,22,28,29,30,31,32,33]. Our theoretical results are in good agreement with these experimental observations. Specifically, for the NOx system, the experimentally reported wavelength associated with $S_{0}\to S_{1}$ is 315 nm, whereas the calculated value is 304 nm. A similar trend was observed for the oscillator strength, with experimental and calculated values of 0.016 and 0.01, respectively. For the NOx-p-NO_2_ system, the experimental wavelength is 313 nm, and the calculated value is 324 nm. Literature reports indicate that electronic rearrangements during vertical transitions involve charge density transfer from the NOx group to the aromatic ring [12,16]. This work analyzes this phenomenon using the electronic redistribution function between the excited ($S_{1}$) and ground ($S_{0}$) states in the NOx, NOx-o-OH, and NOx-o-Cl systems. Figure 1 illustrates this behavior using the isosurfaces of $\Delta_{ij}\rho \left(r\right)=0.04$, complemented by the computed $\langle N_{ij}^{k}\rangle$ values for both the N-O group and aromatic ring. These results provide valuable insights into the charge density redistribution governing the vertical transitions in the studied systems.

The values of $\langle N_{ij}^{k}\rangle$ generally correspond to the reactivity patterns reported in the literature for NOx systems under $S_{0}\to S_{1}$ excitation. For example, the N-O group exhibits electronic density depletion ($\Delta_{ij}\rho \left(r\right)<0$), while the aromatic ring has an electronic density accumulation ($\Delta_{ij}\rho \left(r\right)>0$). Examination of the condensed form of this descriptor over atoms reveals that, within the N-O group, the oxygen atom is responsible for electron density depletion, whereas the nitrogen atom accumulates electronic density. In the aromatic ring, not all carbon atoms gain electronic density; notably, the carbon atom at the *para* position depletes electronic density ($\langle N_{ij}^{k}\rangle <0$). These trends are consistent across all studied systems, including substituted systems such as NOx-o-OH and NOx-o-Cl, which reinforces the reliability of this reactivity description. Analysis of $\Delta_{ij}\rho \left(r\right)$ further elucidates the nature of the interactions between the atoms in the excited states of NOx compounds. The majority of photoproducts generated upon irradiation involve the formation of new C–O bonds. This phenomenon can be rationalized by the charge deficiency on the oxygen atom and the concomitant charge accumulation on the carbon atom upon the $S_{0}\to S_{1}$ transition, as evidenced by the values of $\langle N_{ij}^{k}\rangle$ presented in Figure 1.

**Figure 2:**
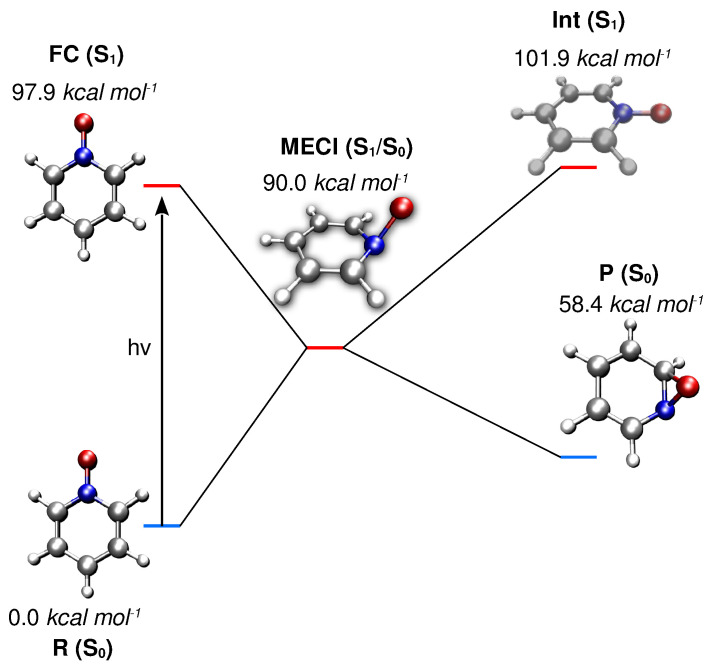
Energy profile of the relevant reaction points involved in the conversion of pyridine N-Oxide into an oxaziridine derivative (7-oxa-1-azabicyclo[4.1.0]hepta-2,4-diene). These include pyridine N-Oxide (*R*), the Franck-Condon point (*FC*), the minimum energy conical intersection (*MECI*), and the oxaziridine-type derivative (*P*). Energies, in kcal mol^−1^, were calculated relative to *R*.

Figure 2 presents the energy diagram for the key energy points involved in the photoinduced electronic rearrangement of NOx compounds. Following the $S_{0}\to S_{1}$ vertical transition, the excited NOx species can decay toward a region where the $S_{1}$ and $S_{0}$ states are degenerate, primarily characterized by the N-O bond distortions. Hence, the minimum energy conical intersection (*MECI*) is 7.9 kcal ${\mathrm{mol}}^{-1}$ below the Franck–Condon (*FC*) point. A higher-energy $S_{1}$ intermediate (*Int*) was also identified at approximately 11.9 kcal mol^−1^ above the *MECI*, characterized by the elongation of the C–C bonds of the aromatic ring without loss of planarity. Products formed from the *MECI* include both an oxaziridine-like intermediate (*P*) and pyridine NOx (*R*), with the latter being more energetically stable.

The main geometrical change observed in NOx during its evolution from the *FC* region to the *MECI* is an out-of-plane rotation of the oxygen atom (O-rotation), characterized by the angle β, which reaches approximately 60º in the *MECI* geometry (see Table 2). Similarly, a significant stretching of the N-O bond was observed, increasing from 1.24 to 1.40 Å, accompanied by a closer approach between the oxygen atom and one of the carbons adjacent to the N-O group. Finally, the decay from the *MECI* to the ground state leading to the formation of *P* involves a reduction in the C-O distance from 2.05 to 1.56 Å, suggesting the formation of this bond. Throughout this process, the angle β remains nearly constant.

The data in Table 3 provide critical insights into the energetic landscape of NOx systems along the *FC* to *MECI* ($\Delta E_{1}=E_{MECI}-E_{FC}$), *MECI* to *Int*($\Delta E_{2}=E_{Int}-E_{MECI}$), and *MECI* to *P* ($\Delta E_{3}=E_{P}-E_{MECI}$) pathways. The *FC* → *MECI* conversion predominantly results in energetic stabilization, as evidenced by consistently negative energy changes. Among the systems studied, NOx-o-OH shows the highest stabilization (−33.8 kcal mol^−1^), highlighting the significant energetic favorability associated with *ortho*-substitution by hydroxyl groups. Conversely, NOx-m-NO_2_ exhibits a destabilizing effect (6.1 kcal mol^−1^). These observations suggest that the O-rotation (from the N-O group) in NOx derivatives, leading to the *MECI* region, contributes to overall stabilization.

In contrast, the energy changes associated with the *MECI* → *Int* conversion revealed a general trend toward destabilization, with positive values observed for most systems. Notably, the NOx-o-NO_2_ and NOx-p-NO_2_ systems exhibit substantially lower destabilization energies (3.4 and 4.1 kcal mol^−1^, respectively), indicating more favorable interactions during the transition to the $S_{1}$ intermediate *Int*. These results further support the idea of a stabilizing effect induced by the O-rotation. This is evidenced by the essentially planar geometry of *Int* and the positive energy associated with the *MECI* → *Int* process, which reflects destabilization.

The return to the ground state, *MECI* → *P*, is characterized by pronounced stabilization for all systems, as reflected by the strongly negative energy changes. The NOx-o-NO_2_ system shows the greatest stabilization (−44.0 kcal mol^−1^), underscoring the exceptional electronic relaxation facilitated by the *ortho*-NO_2_ substituent in the oxaziridine-like formation. By contrast, the NOx-p-OH system exhibits the least stabilization (−32.7 kcal mol^−1^), suggesting that while *para*-hydroxyl substitution provides stabilization, it is less effective in lowering the overall energy.

**Figure 3:**
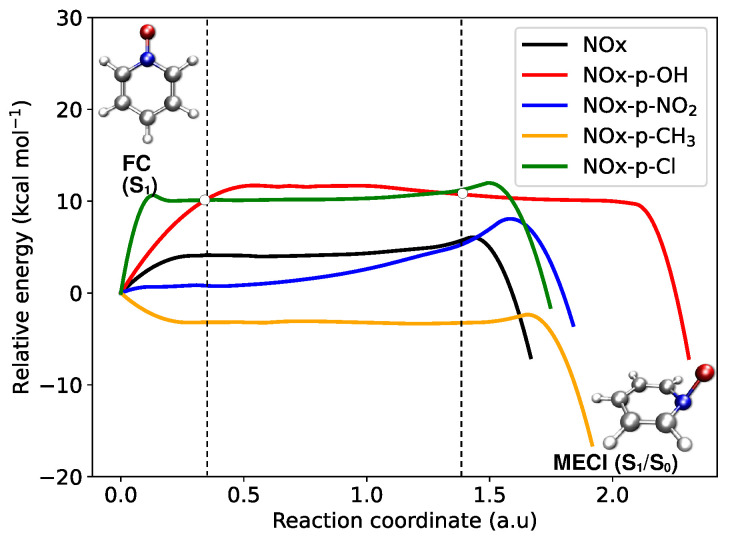
Minimum energy reaction pathway connecting the *FC* and *MECI* points for the *p*-substituted and unsubstituted derivatives. Vertical lines delimit the region of the reaction path corresponding to N-O group rotation. Energies are reported in kcal mol^−1^, and the reaction coordinates are given in Bohr, as calculated using the NEB method.

The influence of substituents is complex, with *ortho*-substitution generally enhancing both stabilization and destabilization effects. Electron-donating groups, such as -OH, tend to promote significant stabilization along the *FC* → *MECI* and *MECI* → *P* transitions. In contrast, electron-withdrawing groups such as -NO_2_ show a more nuanced behavior, offering pronounced stabilization in the *MECI* → *P* deactivation while occasionally destabilizing early transitions. Although the reaction pathways *FC* → *MECI* and *MECI* → *P* stabilize the system in the excited state $S_{1}$, it is necessary to assess the potential existence of reaction barriers in these processes. Figure 3 illustrates the minimum-energy reaction pathway *FC* → *MECI* of the NOx systems with *p*-substitution.

The deactivation of NOx following the *FC* → *MECI* transition involves energy barriers of approximately 10.0 kcal mol^−1^, which is predominantly observed in the NOx-p-OH and NOx-p-Cl systems (see Figure 3). In contrast, systems such as NOx, NOx-p-NO_2_, and NOx-p-Cl exhibit smaller or negligible barriers. These barriers are not primarily associated with O-rotation but rather result from distortions of the aromatic ring. The O-rotation maintains a stable energy profile, forming a plateau along the reaction coordinate, followed by a sharp energy decrease as the system approaches the *MECI*.

**Figure 4:**
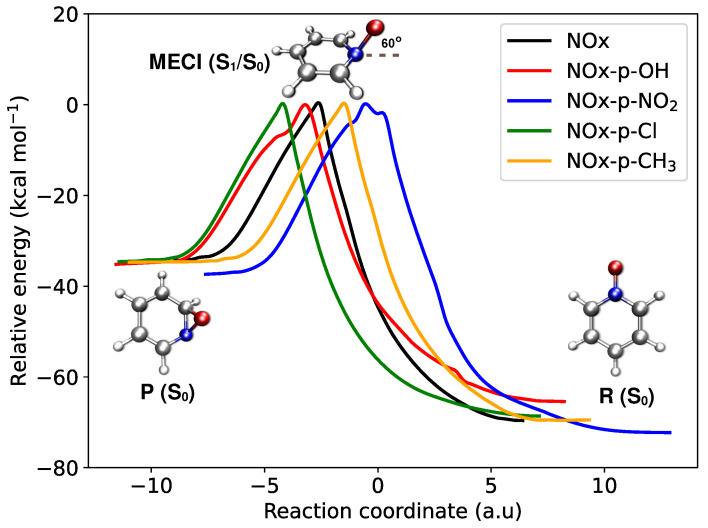
Minima energy reaction pathways connecting the *MECI* → R and *MECI* → *P* points for the *p*-substituted derivatives and the unsubstituted derivative correspond to deactivation towards the ground state. Energies, in kcal mol^−1^, and the reaction coordinates, in dimensionless units, are calculated using the IRC method.

The formation of photoproducts (*R* and *P*) through *MECI* does not involve an energy barrier in the ground state, particularly regarding the return to *R*. Furthermore, the generation of oxaziridine-like derivatives was energetically favorable, with the most significant effect observed in systems substituted with $NO_{2}$. The *MECI* → *R* reaction pathway suggests that the planarity of the NOx ring is preferentially stabilized in the ground state. In contrast, the *MECI* → *P* pathway illustrates that the formation of the O-C bond leading to the oxaziridine-like photoproduct is energetically favored (see Figure 4). Let us recall that, although oxaziridine-like products are formed from the *MECI*, they correspond to intermediates that precede the formation of more stable products, such as 1,2-oxazepines. In this study, pyridine NOx was used as a structural motif to explore the reaction pathways leading to the formation of oxaziridines and, potentially, oxazepine-like structures. Although the conversion of pyridine N-Oxides into 1,2-oxazepines has not been experimentally confirmed, it has been reported that analogous systems with larger aromatic frameworks, such as acridine N-Oxides, can undergo such rearrangements [19]. Using pyridine NOx as a model allows for the analysis of electronic and structural factors that may influence the feasibility of these processes in larger systems.

**Figure 5:**
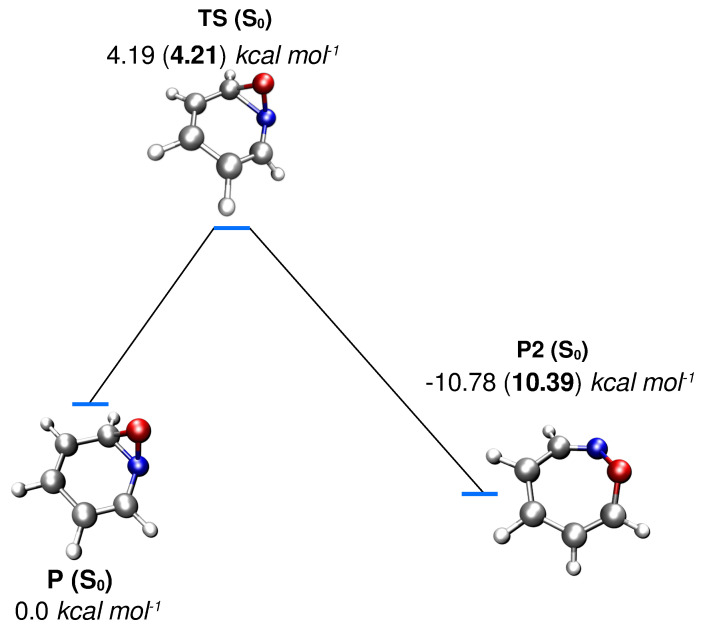
Energy profile for key stationary points along the oxaziridine → 1,2-oxazepine conversion. The oxaziridine intermediate (*P*), the transition state (*TS*), and the 1,2-oxazepine derivative (*P*2) are depicted. Energies, in kcal mol^−1^, were calculated relative to *P* in ethanol and benzene, with the values of the former solvent in parentheses.

The interconversion of these products is governed by the polarity of the solvent. Figure 5 depicts the reaction scheme for the conversion of oxaziridine-like derivatives into the corresponding 1,2-oxazepines, along with the reaction barriers in solvents, such as benzene and ethanol.

The *P* → *P*2 reaction pathway involves a relatively low energy barrier of approximately 4.2 kcal mol^−1^ in both solvents, explaining the facile interconversion between these species, as previously reported experimentally. However, the formation of the 1,2-oxazepine product is about 10.8 kcal mol^−1^ lower in energy than the oxaziridine derivative, indicating greater thermodynamic stability. In addition, the barrier associated with the P→P2 step is characterized by the transition state (*TS*), where the N-C bond length is 1.73 Å, elongating to 2.30 Å as the 1,2-oxazepine derivative forms. Table 4 summarizes the energy changes associated with the P→TS ($\Delta E_{4}=E_{\mathrm{TS}}-E_{P}$) and P→P2 ($\Delta E_{5}=E_{P2}-E_{P}$) processes for the NOx-substituted systems. A notably low reaction barrier was observed for the conversion of the oxaziridine-like intermediate to 1,2-oxazepine, with the barrier disappearing for NO_2_-substituted derivatives, where $\Delta E_{4}<0$. These results further confirm the greater stability of the 1,2-oxazepine derivatives compared to their oxaziridine counterparts, suggesting that substitutions on the NOx ring might not significantly affect this behavior. Analysis of the solvent effects reveals that the activation barriers (*P* → *TS*) are generally lower in ethanol than in benzene, indicating greater *TS* stabilization in the polar medium. This implies that the formation of 1,2-oxazepine derivatives is favored in this solvent, consistent with previous reports [12,23]. Electron-donating groups, such as CH_3_ and OH, tend to reduce the activation barriers, particularly at the -*o* and -*m* positions. In contrast, electron-withdrawing groups, such as NO_2_, significantly lower the barriers in both solvents, making the reactions highly favorable, especially in ethanol. Substituents such as Cl lead to higher barriers, particularly in benzene, reflecting weaker interactions between the system and the nonpolar solvent.

Although the differences in energy barriers between ethanol and benzene provide insight into the solvent influence on the oxaziridine-like derivatives and their conversion to the corresponding 1,2-oxazepine, these differences are not substantial. Therefore, a more comprehensive discussion of solvent effects is warranted. Such a discussion would require explicit inclusion of solvent effects in the calculations. In addition to the energetic implications associated with the excited-state electronic rearrangements that NOx systems may undergo, we also focused on analyzing the chemical bonding involved in this process, particularly during the stages spanning from the reactants to the *MECI* region. To this end, we calculated the average number of electrons in the chemically relevant regions obtained through ELF partitioning, namely, chemical bond and non-bonding electron pairs. This approach enables the examination of electronic population evolution at relevant structures (*R*, *FC*, and *MECI*), identifying bond formation and cleavage, and electron pair rearrangements.

**Figure 6:**
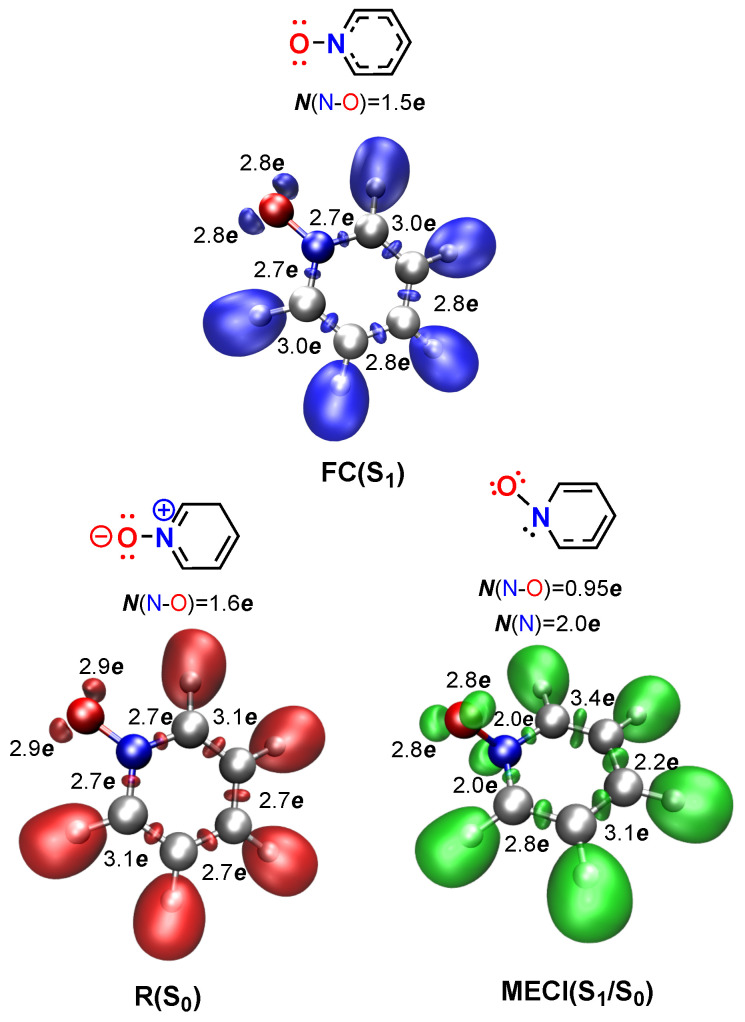
Isosurfaces of the electron localization function (ELF) (η(r)=0.99) for *R*, *MECI*, and *FC*. The calculated bond and lone pair populations are shown. Lewis-like structures derived from ELF analysis are presented.

**Figure 7:**
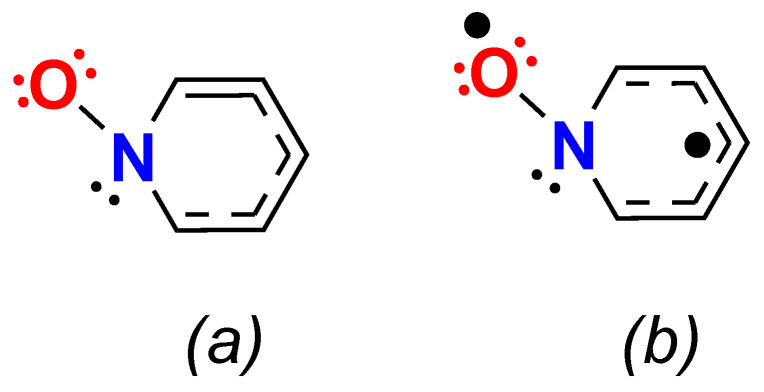
Proposed Lewis-like structure for *MECI* associated with pyridine N-Oxide derived from through chemical bonding analysis of the electron localization function (**a**); Lewis-like structure typically used in the literature to explain the photochemistry of N-Oxides (**b**).

Figure 6 displays the ELF isosurfaces, electronic populations for C-C, C-N, and N-O bonds, and non-bonding regions associated with N and O at the *R*, *FC*, and *MECI* points. Additionally, Lewis-like representations based on the ELF analysis were included for each point, providing a complementary visual interpretation of the chemical bonding throughout the studied process. The $S_{0}\to S_{1}$ excitation process induces significant changes in the electronic distribution of the bonds. An increase of 0.1**e** is observed in the electronic population of the C-C bonds farthest from the N-O group (C2-C3 and C3-C4), whereas the N-O bond weakens as its electronic population decreases by 0.1**e**. These electronic rearrangements have been previously reported in the literature, reinforcing the reliability of our ELF analysis in describing this behavior. Furthermore, it is noteworthy that the C1-C2 and C2-C3 bonds also weaken, with a reduction of 0.1**e** in their electronic population as a consequence of $S_{0}\to S_{1}$ excitation. During the *FC* → *MECI* decay, the electronic rearrangements were more substantial. The N-C bonds near the N-O group transform into single bonds, with their populations decreasing from 2.7 to 2.0**e**. In contrast, double bonds C=C begin to form, defining the structure of the oxazirine-like derivative, as seen in the increasing populations of the C2-C3 and C4-C5 bonds, which reach 3.1**e** and 3.4**e**, respectively. The N-O bond became significantly weaker, with its population dropping to 0.95**e**. Simultaneously, a non-bonding region emerged on the nitrogen atom, resembling the lone pairs in NH_3_. This region accumulated at 2.0**e**, confirming the formation of lone pairs over the nitrogen atom. Notably, ELF analysis reveals the absence of pairing density between the oxygen atom in the N-O group and the adjacent carbons. This indicates that the C-O bond responsible for the oxaziridine ring formation does not occur on the excited-state $S_{1}$ surface. Instead, this bond appears only after the *MECI* deactivates toward the ground state. Unlike previous studies on chemical bonding, the literature has documented an intermediate [12,16] with a Lewis structure similar to that of the *MECI* identified in this study, as shown in Figure 7.

The structure proposed in the literature suggests the presence of radical centers on both the ring and the oxygen atom. However, the ELF analysis unveils that no non-bonding regions are present on the oxygen atom beyond those associated with its lone pairs. While previous studies indicate the presence of 1.0**e** delocalized over the pyridine ring, our results suggest that although there is an increase in the electronic populations of the C-C bonds, one of these bonds in the pyridine ring has already acquired a double-bond character, with a population of 3.4**e**. According to ELF analysis, this population is consistent with the formation of double bonds [34]. It is important to note that these observations hold for all systems studied, including all substitutions. The electronic populations reflecting this behavior are detailed in the Supporting Information (SI).

## 3. Materials and Methods

**Figure 8:**
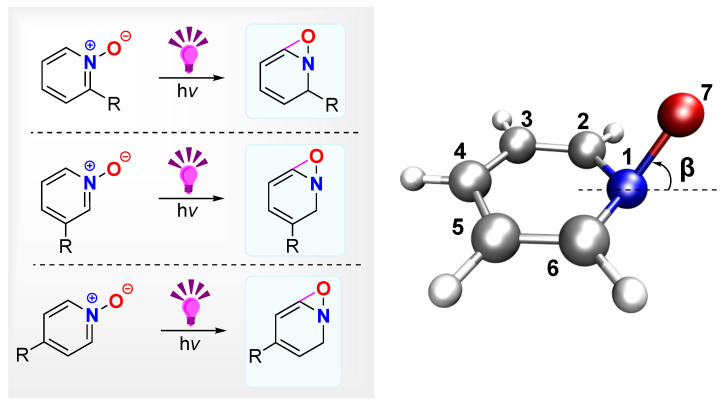
N-Oxide pyridine systems used in the present study indicate the types of substitutions considered (*R* = H, CH_3_, Cl, NO_2_, OH) and the reactions analyzed. Additionally, a 3D model was included, showing the atom numbering employed throughout the manuscript, as well as the angle β, representing the out-of-plane rotation of the oxygen atom in the N-O bond.

The lowest-energy excited singlet state, $S_{1}$, was used to investigate the photoinduced rearrangement of pyridine N-Oxides [16]. Monosubstituted pyridine N-Oxides at the ortho, meta, and para positions with R = H, CH_3_, Cl, NO_2_, and OH substituents were analyzed, as illustrated in Figure 8. Electronic structure calculations were performed using Gaussian16 [35] and ORCA 5.0 [36] computational packages, employing ab initio methods based on the Complete Active Space Self-Consistent Field (CASSCF) [37] approach. The active space used consisted of ten electrons distributed in eight orbitals along with the cc-pVDZ basis set [38]. The active space used in this study, composed of ten active orbitals and eight reference orbitals (10,8), includes the π orbitals of the pyridine ring, the N-O bonding orbital, as well as a pair of non-bonding orbitals of oxygen. It is worth mentioning that larger active spaces, such as (12,10) and (14,12), were tested, but no significant changes were observed in the excitation energies or the composition of the states; therefore, the CAS(10,8) space was selected. Refer to the SI for accessing the optimized Cartesian geometries of relevant structures, and details on excitation energies of $S_{0}$ → $S_{n}$, relative energies characterizing the *FC* and *MECI* transitions, and electronic populations of bonds. Solvent effects were considered using the conductor-like polarizable continuum model (CPCM) [39] for reactions occurring only in the ground state, specifically for the conversion of the oxaziridine-type intermediate into 1,2-oxazepine derivatives. In contrast, solvent effects were omitted for the excited state, as there were no appreciable energy differences observed when including solvation (variations of approximately ±2.4 kcal mol^−1^). The optimization of energetically relevant points, such as minima, intermediates, and minimum energy conical intersection (*MECI*) points, was carried out using a state-averaged scheme (SA-CASSCF).

Minimum energy paths connecting relevant points were mapped using nudged elastic band (NEB) [40] and intrinsic reaction coordinate (IRC) [41] calculations. The energies of all relevant reaction structures, including minima, transition states, and conical intersections, were corrected using the NEVPT2 method to include dynamic correlation. Chemical bonding analysis was performed using the electron localization function (ELF) [42] with natural orbitals [43] derived from the CAS(10,8) wavefunction. ELF is particularly valuable for understanding the formation and dissociation of chemical bonds by analyzing the electron pair representation within the ELF topology [44]. In this context, ELF basins, which represent the core, bonding, and nonbonding regions, are distinct from atomic subsystems. The ELF method is essential for experimental and theoretical studies of complex bonding, particularly in the context of electron pair reorganizations [45]. The ELF field is examined in a three-dimensional space by defining *f*-localization domains, which are bounded by isosurfaces, where η(r)=f. These domains are classified as irreducible (containing a single attractor) or reducible (containing multiple attractors). The gradient of the ELF function identifies critical points, which are analyzed using the Hessian matrix [46]. Attractors are maxima in the η(r) field and are related to key concepts in chemical bonding, such as lone pairs, bonds, and the distinction between valence and core shells. The ELF topology distinguishes between core attractors, C(C), and valence attractors, V(A,B), with basins defined by the paths leading to an attractor. Synaptic order quantifies the number of core basins connected to a valence basin. Monosynaptic basins are associated with lone pairs, disynaptic basins with bonds, and asynaptic basins with no connection to core basins. For example, in the NOx molecule, the disynaptic basins represent the C-C, N-O, N-C, and N-H bonds, whereas the monosynaptic basins represent the lone pairs on the oxygen atom. The electron population within ELF basins provides valuable insights into the nature of the bond, typically showing values of approximately two electrons for single bonds and four electrons for double bonds. For simplicity, we will refer to bond populations and lone pair populations rather than using basin notation to make the discussion more aligned with chemical language. This topological analysis has been extensively applied to the study of various reaction mechanisms, including cycloadditions [47,48], photoreactions [49,50], hemicellulose pyrolysis [51], and developing a framework for scaling bonds polarity [52].

Additionally, the redistribution of the electronic density due to $S_{0}\to S_{1}$ excitation was analyzed using the following function:

(1) $$\Delta_{ij}\rho \left(r\right)=c_{j}^{2}\rho_{j}\left(r\right)-c_{i}^{2}\rho_{i}\left(r\right)$$

This function allows the identification of reactive sites in a molecular system in the excited state *j* compared to the state *i* (i.e., $S_{1}$ and $S_{0}$, respectively). The coefficients $c_{i}$ and $c_{j}$ were determined from the CI expansion of the CAS. This redistribution can be condensed at atomic sites $\Omega_{k}$ by partitioning the average density of states and integrating $\Delta_{ij}\rho \left(r\right)$:

(2) $$\langle N_{ij}^{k}\rangle =\int_{\Omega_{k}}\Delta_{ij}\rho \left(r\right)\;dr$$

The values $\langle N_{ij}^{k}\rangle <0$ and $\langle N_{ij}^{k}\rangle >0$ indicate, respectively, the deficiency and excess of charge density of state *j* compared to *i* at atomic site *k*. In this study, we employed the Hirshfeld partitioning scheme to obtain $\langle N_{ij}^{k}\rangle$ We employed this approach to characterize the excited-state reactivity of tropones [53].

## 4. Conclusions

The photochemical activation of pyridine N-Oxides and their substituted derivatives reveals a complex mechanistic landscape marked by intricate excited-state distortions and electronic rearrangements. Our study demonstrated that deactivation of the *S*_1_ excited state, driven by the out-of-plane rotation of the N-O oxygen atom and significant charge redistribution, facilitates the formation of oxaziridine-like intermediates. This process involved a pronounced weakening of the N-O bond and the formation of a lone pair on the nitrogen atom. Simultaneously, a C=C double bond, characteristic of the oxaziridine-like intermediate, emerged in the S_1_ excited state. These intermediates showed a favorable ground-state transformation into 1,2-oxazepine derivatives, with energy barriers influenced by solvent polarity and substituent effects. Electron-donating groups (-OH) stabilize both excited-state relaxation and ground-state rearrangement, while electron-withdrawing groups (-NO_2_) enhance product stabilization. Polar solvents further facilitate interconversion. These insights advance the understanding of pyridine N-Oxide photochemistry, guiding the design of photoreactive systems and synthetic strategies.

## Figures and Tables

**Table 1:** Excitation data for the $S_{0}\to S_{1}$ transition of various NOx systems and their derivatives (rounded Foc values).

System	Wavelength (nm)	Energy (eV)	Focs
NOx	304	4.08	0.01
NOx m-CH_3_	285	4.35	0.02
NOx o-CH_3_	290	4.27	0.01
NOx p-CH_3_	296	4.19	0.01
NOx m-Cl	301	4.11	0.01
NOx o-Cl	301	4.12	0.03
NOx p-Cl	318	3.90	0.01
NOx m-NO_2_	327	3.79	0.03
NOx o-NO_2_	296	4.19	0.03
NOx p-NO_2_	290	4.27	0.02
NOx m-OH	286	4.33	0.03
NOx o-OH	278	4.46	0.04
NOx p-OH	324	3.82	0.04

**Table 2:** Geometric parameters of relevant structures associated with the conversion of pyridine N-Oxide to the oxaziridine derivative. The N-O, C-O, and N-C bond distances (in Ångstrom, Å) and the rotation angle β are included.

System	N-O (Å)	C-O (Å)	N-C (Å)	β
R($S_{0}$)	1.24	2.27	1.36	0
Int($S_{1}$)	1.29	2.31	1.38	0.02
MECI($S_{1}/S_{0}$)	1.40	2.05	1.39	59.35
P($S_{1}$)	1.38	1.59	1.42	53.80

**Table 3:** Relative energies (in kcal mol^−1^) of substituted pyridine N-Oxides for relevant structures involved in the production of oxaziridine-like derivatives. The analysis includes the transitions from *FC* to *MECI* ($\Delta E_{1}$), *Int* to *MECI* ($\Delta E_{2}$), and *MECI* to *P* ($\Delta E_{3}$).

System	$\Delta E_{1}$	$\Delta E_{2}$	$\Delta E_{3}$
NOx	−7.00	11.04	−36.05
NOx-m-CH_3_	−18.14	10.67	−34.31
NOx-o-CH_3_	−21.92	13.60	−31.45
NOx-p-CH_3_	−16.53	13.27	−34.37
NOx-m-Cl	−17.77	14.55	−35.60
NOx-o-Cl	−25.01	15.15	−35.41
NOx-p-Cl	−1.53	11.60	−36.49
NOx-m-NO_2_	6.08	10.50	−35.71
NOx-o-NO_2_	−4.79	3.39	−43.95
NOx-p-NO_2_	−3.48	4.11	−41.62
NOx-m-OH	−13.79	8.17	−36.06
NOx-o-OH	−33.79	15.98	−37.37
NOx-p-OH	−7.07	18.87	−32.72

**Table 4:** Relative energies of the conversion reaction of oxaziridine-type intermediates to 1,2-oxazepines (*P* → *P*2) and the corresponding energy barriers (*P* → *TS*) in ethanol and benzene solvents.

System	Ethanol		Benzene	
	$\Delta E_{4}$	$\Delta E_{5}$	$\Delta E_{4}$	$\Delta E_{5}$
Nox	4.2	−10.8	4.2	−10.4
Nox-m-CH_3_	4.0	−10.1	5.1	−9.8
Nox-o-CH_3_	3.6	−10.9	3.9	−11.5
Nox-p-CH_3_	4.8	−10.7	4.0	−10.3
Nox-m-Cl	0.6	−15.0	1.6	−17.7
Nox-o-Cl	12.1	−12.5	7.1	−9.0
Nox-p-Cl	2.1	−13.6	0.5	−15.4
Nox-m-NO_2_	−12.2	−15.6	−1.8	−24.1
Nox-o-NO_2_	−6.4	−8.2	−5.9	−14.7
Nox-p-NO_2_	−6.5	−16.7	−4.8	−8.2
Nox-m-OH	5.6	−9.5	3.6	−9.7
Nox-o-OH	7.2	−11.5	5.3	−8.9
Nox-p-OH	1.5	−13.2	1.1	−12.9

## Data Availability

The original contributions presented in this study are included in the article/Supplementary Materials. Further inquiries can be directed to the corresponding authors.

## Supplementary Materials

The following supporting information can be downloaded at: https://www.mdpi.com/article/10.3390/molecules30244776/s1. Optimized Cartesian coordinates, intrinsic reaction coordinates, excitation energies, and electron populations.
