# Supplementary material for: Photochemical Rearrangements of Pyridine N-Oxides: Pathways to Oxaziridine Derivatives
Source: Molecules. 2025 Dec 14;30(24):4776. doi: 10.3390/molecules30244776 (PMC12736228; doi:10.3390/molecules30244776)
Supplement: Supplementary file 1 [file molecules-30-04776-s001.zip › SupportingInformation_Molecules-3997391_Revision1-R1.pdf]

Supporting Information

Photochemical Rearrangements of Pyridine N-Oxides: Pathways to Oxaziridine Derivatives

Cristian J. Guerra [1,*] , Yeray A. Rodríguez-Núñez [1] , Efraín Polo-Cuadrado [2] , Mitchell Bacho [3], Jorge Soto-Delgado [4] , Victor B. Fuentes-Guerrero [1], Eduardo I. Torres-Olguín [1] , Cristopher A. Fica-Cornejo [1] , Daniela Rodríguez-García [1] , Manuel E. Taborda-Martínez [5], Leandro Ayarde-Henríquez [6,7,*] and Adolfo E. Ensuncho [8]

1 Laboratorio de Síntesis y Reactividad de Compuestos Orgánicos, Departamento de Ciencias Químicas, Facultad de Ciencias Exactas, Universidad Andrés Bello, República 275, Santiago 8370146, Chile

2 Laboratorio de Diseño y Síntesis de Compuestos Bioactivos, Departamento de Química Orgánica, Facultad de Ciencias Químicas, Universidad de Concepción, Concepción 4070386, Chile

3 Departamento de Ciencias Biológicas y Químicas, Facultad de Medicina y Ciencia, Universidad San Sebastián, Campus los Leones, Lota 2465, Providencia, Santiago 7510602, Chile

4 Departamento de Ciencias Químicas, Facultad de Ciencias Exactas Bello, Universidad Andrés Bello, Quillota 980, Viña del Mar 2531015, Chile

5 Facultad de Ciencias de la Educación, Universidad del Magdalena, Carrera 32 No. 22-08, Santa Marta 470004, Colombia

6 School of Physics, Trinity College Dublin, 2 D02 PN40 Dublin, Ireland

7 AMBER, Advanced Materials and BioEngineering Research Centre, 2 D02 PN40 Dublin, Ireland

8 Grupo de Química Computacional, Facultad de Ciencias Básicas, Universidad de Córdoba, Carrera 6 No. 77-305, Montería-Córdoba 230001, Colombia

* Correspondence: c.guerramadera@uandresbello.edu (C.J.G.); leandro.ayarde@tcd.ie (L.A-H.)

Contents

List of Tables

Table S1 $\Delta E = E(S_1) - E(S_0)$ in eV at CAS(10,8) and CAS(14,12). MECI(S_1/S_0) was optimized at CAS(10,8)/cc-pVTZ. 3
Table S2 Relative energies (kcal mol^{-1}) for transitions involving the *Int* intermediate on the S_1 surface. 3
Table S3 Wavelengths, excitation energies, and oscillator strengths for $S_0 \to S_n$ transitions in the studied N-oxides. 3
Table S4 Wavelengths, excitation energies, and oscillator strengths for $S_0 \to S_n$ tran-sitions in the studied N-oxides, using different active spaces such as (10e, 8o), (12e, 10o), and (14e, 12o). 5
Table S5 Relative energies (kcal mol^{-1}) for the substituted pyridine N-oxides along the stationary energy points characterizing the FC to MECI transitions, calculated in gas phase and ethanol. 6
Table S6 Electronic populations calculated over bonds and non-bonding regions using the partitioning provided by the ELF for the FC point in all studied systems. . . . 7
Table S7 Electronic populations calculated over bonds and non-bonding regions using the partitioning provided by the ELF for the MECI point in all studied systems. . . 7

List of Figures

Figure S1 Minimum energy reaction pathway connecting the Int $\to$ MECI for the *p*-substituted derivatives. The energies are shown in kcal mol^{-1}, and the reaction coordinates are given in dimensionless units calculated using the IRC method. . . . 8

Table S1: $\Delta E = E(S_1) - E(S_0)$ in eV at CAS(10,8) and CAS(14,12). MECI(S_1/S_0) was optimized at CAS(10,8)/cc-pVTZ.

**System**	CAS(10,8)	CAS(14,12)
NOx	0.001	0.293
NOx-m-CH_3	0.004	0.268
NOx-o-CH_3	0.002	0.304
NOx-p-CH_3	0.001	0.096
NOx-m-Cl	0.005	0.153
NOx-o-Cl	0.004	0.198
NOx-p-Cl	0.005	0.321
NOx-m-NO_2	0.072	0.128
NOx-o-NO_2	0.001	0.010
NOx-p-NO_2	0.093	0.141
NOx-m-OH	0.008	0.112
NOx-o-OH	0.002	0.068

Table S2: Relative energies (kcal mol^{-1}) for transitions involving the *Int* intermediate on the S_1 surface.

**System**	ΔE_A	ΔE_B	ΔE_C
NOx	-7.00	11.04	4.04
NOx-m-CH_3	-18.14	10.67	-7.47
NOx-o-CH_3	-21.92	13.60	-8.32
NOx-p-CH_3	-16.53	13.27	-3.26
NOx-m-Cl	-17.77	14.55	-3.22
NOx-o-Cl	-25.01	15.15	-9.86
NOx-p-Cl	-1.53	11.60	10.07
NOx-m-NO_2	6.08	10.50	16.58
NOx-o-NO_2	-4.79	3.39	-1.40
NOx-p-NO_2	-3.48	4.11	0.63
NOx-m-OH	-13.79	8.17	-5.62
NOx-o-OH	-33.79	15.98	-17.81
NOx-p-OH	-7.07	18.87	11.80

Table S3: Wavelengths, excitation energies, and oscillator strengths for $S_0 \rightarrow S_n$ transitions in the studied N-oxides.

**System**	**Excitation**	**Wavelength (nm)**	**Energy (eV)**	**Focs**
NOx	$S_0 \rightarrow S_1$	304	4.08	0.00650
	$S_0 \rightarrow S_2$	229	5.41	0.31372
	$S_0 \rightarrow S_3$	194	6.39	0.24428
	$S_0 \rightarrow S_4$	187	6.63	0.08451
NOx m-CH_3	$S_0 \rightarrow S_1$	285	4.35	0.02301
	$S_0 \rightarrow S_2$	256	4.84	0.00001
	$S_0 \rightarrow S_3$	228	5.45	0.33200

System	Excitation	Wavelength (nm)	Energy (eV)	Focs
	$S_0 \rightarrow S_4$	226	5.48	0.00094
NOx o-CH_3	$S_0 \rightarrow S_1$	290	4.27	0.01121
	$S_0 \rightarrow S_2$	242	5.13	0.27155
	$S_0 \rightarrow S_3$	189	6.58	0.29765
	$S_0 \rightarrow S_4$	170	7.29	0.39871
NOx p-CH_3	$S_0 \rightarrow S_1$	296	4.19	0.00880
	$S_0 \rightarrow S_2$	250	4.96	0.47232
	$S_0 \rightarrow S_3$	184	6.72	0.21679
	$S_0 \rightarrow S_4$	174	7.13	0.04703
NOx m-Cl	$S_0 \rightarrow S_1$	301	4.11	0.00854
	$S_0 \rightarrow S_2$	245	5.07	0.28853
	$S_0 \rightarrow S_3$	192	6.45	0.27778
	$S_0 \rightarrow S_4$	174	7.12	0.36181
NOx o-Cl	$S_0 \rightarrow S_1$	301	4.12	0.03046
	$S_0 \rightarrow S_2$	274	4.53	0.00001
	$S_0 \rightarrow S_3$	242	5.13	0.00039
	$S_0 \rightarrow S_4$	232	5.34	0.32712
NOx p-Cl	$S_0 \rightarrow S_1$	318	3.90	0.01143
	$S_0 \rightarrow S_2$	244	5.07	0.48222
	$S_0 \rightarrow S_3$	188	6.60	0.18413
	$S_0 \rightarrow S_4$	177	7.02	0.03881
NOx m-NO_2	$S_0 \rightarrow S_1$	327	3.79	0.02999
	$S_0 \rightarrow S_2$	241	5.14	0.52106
	$S_0 \rightarrow S_3$	187	6.61	0.00424
	$S_0 \rightarrow S_4$	168	7.39	0.00392
NOx o-NO_2	$S_0 \rightarrow S_1$	296	4.19	0.02838
	$S_0 \rightarrow S_2$	259	4.79	0.23916
	$S_0 \rightarrow S_3$	180	6.89	0.00256
	$S_0 \rightarrow S_4$	163	7.61	0.03125
NOx p-NO_2	$S_0 \rightarrow S_1$	290	4.27	0.01737
	$S_0 \rightarrow S_2$	275	4.51	0.57883
	$S_0 \rightarrow S_3$	204	6.09	0.00786
	$S_0 \rightarrow S_4$	175	7.08	0.09733
NOx m-OH	$S_0 \rightarrow S_1$	286	4.33	0.03429
	$S_0 \rightarrow S_2$	272	4.56	0.00002
	$S_0 \rightarrow S_3$	228	5.43	0.35107
	$S_0 \rightarrow S_4$	216	5.73	0.00055
NOx o-OH	$S_0 \rightarrow S_1$	278	4.46	0.03552
	$S_0 \rightarrow S_2$	260	4.78	0.00002
	$S_0 \rightarrow S_3$	231	5.37	0.34475
	$S_0 \rightarrow S_4$	218	5.70	0.00112
NOx p-OH	$S_0 \rightarrow S_1$	324	3.82	0.04439
	$S_0 \rightarrow S_2$	257	4.83	0.00002
	$S_0 \rightarrow S_3$	246	5.04	0.00041
	$S_0 \rightarrow S_4$	232	5.34	0.43326

Table S4: Wavelengths, excitation energies, and oscillator strengths for $S_0 \rightarrow S_n$ transitions in the studied N-oxides, using different active spaces such as (10e, 8o), (12e, 10o), and (14e, 12o).

**System**	**Excitation**	**Wavelength (nm)**	**Energy (eV)**	**Focs**
NOx (10e,8o)	$S_0 \rightarrow S_1$	307	4.04	0.02221
	$S_0 \rightarrow S_2$	289	4.29	0.00000
	$S_0 \rightarrow S_3$	251	4.94	0.00021
	$S_0 \rightarrow S_4$	246	5.04	0.32073
NOx (12e,10o)	$S_0 \rightarrow S_1$	307	4.04	0.01143
	$S_0 \rightarrow S_2$	250	4.96	0.30536
	$S_0 \rightarrow S_3$	189	6.55	0.17666
	$S_0 \rightarrow S_4$	173	7.15	0.00907
NOx (14e,12o)	$S_0 \rightarrow S_1$	307	4.03	0.02315
	$S_0 \rightarrow S_2$	286	4.33	0.00000
	$S_0 \rightarrow S_3$	246	5.03	0.00019
	$S_0 \rightarrow S_4$	241	5.14	0.32719
NOx-p-CH_3 (10e,8o)	$S_0 \rightarrow S_1$	281	4.42	0.00646
	$S_0 \rightarrow S_2$	251	4.94	0.48861
	$S_0 \rightarrow S_3$	181	6.86	0.13552
	$S_0 \rightarrow S_4$	179	6.92	0.19080
NOx-p-CH_3 (12e,10o)	$S_0 \rightarrow S_1$	277	4.47	0.00600
	$S_0 \rightarrow S_2$	250	4.96	0.50250
	$S_0 \rightarrow S_3$	180	6.88	0.16681
	$S_0 \rightarrow S_4$	178	6.95	0.18537
NOx-p-CH_3 (14e,12o)	$S_0 \rightarrow S_1$	277	4.47	0.00599
	$S_0 \rightarrow S_2$	245	5.06	0.52109
	$S_0 \rightarrow S_3$	181	6.87	0.18308
	$S_0 \rightarrow S_4$	177	7.01	0.17159
NOx-p-Cl (10e,8o)	$S_0 \rightarrow S_1$	304	4.08	0.01183
	$S_0 \rightarrow S_2$	241	5.14	0.45782
	$S_0 \rightarrow S_3$	184	6.75	0.27442
	$S_0 \rightarrow S_4$	168	7.38	0.44025
NOx-p-Cl (12e,10o)	$S_0 \rightarrow S_1$	315	3.93	0.01004
	$S_0 \rightarrow S_2$	243	5.10	0.49259
	$S_0 \rightarrow S_3$	185	6.70	0.18452
	$S_0 \rightarrow S_4$	175	7.07	0.04356
NOx-p-Cl (14e,12o)	$S_0 \rightarrow S_1$	316	3.92	0.00611
	$S_0 \rightarrow S_2$	233	5.33	0.33191
	$S_0 \rightarrow S_3$	203	6.10	0.30288
	$S_0 \rightarrow S_4$	187	6.65	0.07554
NOx-p-NO_2 (10e,8o)	$S_0 \rightarrow S_1$	290	4.27	0.01779
	$S_0 \rightarrow S_2$	275	4.51	0.58018
	$S_0 \rightarrow S_3$	204	6.09	0.00788
	$S_0 \rightarrow S_4$	175	7.08	0.09707
NOx-p-NO_2 (12e,10o)	$S_0 \rightarrow S_1$	288	4.31	0.01524
	$S_0 \rightarrow S_2$	273	4.54	0.53132
	$S_0 \rightarrow S_3$	200	6.20	0.00185

System	Excitation	Wavelength (nm)	Energy (eV)	Focs
	$S_0 \rightarrow S_4$	175	7.08	0.07428
NOx-p-NO_2 (14e,12o)	$S_0 \rightarrow S_1$	306	4.05	0.00256
	$S_0 \rightarrow S_2$	250	4.97	0.22229
	$S_0 \rightarrow S_3$	213	5.82	0.00706
	$S_0 \rightarrow S_4$	177	7.00	0.02245
NOx-p-OH (10e,8o)	$S_0 \rightarrow S_1$	306	4.05	0.04095
	$S_0 \rightarrow S_2$	247	5.02	0.00002
	$S_0 \rightarrow S_3$	240	5.16	0.00086
	$S_0 \rightarrow S_4$	229	5.40	0.36941
NOx-p-OH (12e,10o)	$S_0 \rightarrow S_1$	334	3.71	0.04424
	$S_0 \rightarrow S_2$	274	4.53	0.00002
	$S_0 \rightarrow S_3$	260	4.78	0.00030
	$S_0 \rightarrow S_4$	244	5.09	0.33328
NOx-p-OH (14e,12o)	$S_0 \rightarrow S_1$	335	3.70	0.04690
	$S_0 \rightarrow S_2$	274	4.53	0.00008
	$S_0 \rightarrow S_3$	256	4.84	0.00040
	$S_0 \rightarrow S_4$	240	5.18	0.30627

Table S5: Relative energies (kcal mol^{-1}) for the substituted pyridine N-oxides along the stationary energy points characterizing the FC to MECI transitions, calculated in gas phase and ethanol.

System	ΔE_1 (ethanol)	ΔE_1 (gas)
NOx	-6.00	-7.00
NOx-m-CH_3	-21.50	-18.14
NOx-o-CH_3	-24.15	-21.92
NOx-p-CH_3	-19.29	-16.53
NOx-m-Cl	-20.72	-17.77
NOx-o-Cl	-27.28	-25.01
NOx-p-Cl	-4.33	-1.53
NOx-m-NO_2	4.51	6.08
NOx-o-NO_2	-10.49	-4.79
NOx-p-NO_2	-6.57	-3.48
NOx-m-OH	-20.62	-13.79
NOx-o-OH	-38.39	-33.79
NOx-p-OH	-12.71	-7.07

Table S6: Electronic populations calculated over bonds and non-bonding regions using the parti-tioning provided by the ELF for the FC point in all studied systems.

**System**	**C1-N6**	**C1-C2**	**C2-C3**	**C3-C4**	**C4-C5**	**C5-N**	**N-O**	**O1**	**O2**	**N6**
NOx	2.68	3.11	2.74	2.73	3.11	2.66	1.60	2.92	2.93	0.00
NOx-m-CH_3	2.56	3.13	2.75	2.89	3.12	2.76	1.62	2.81	2.96	0.00
NOx-o-CH_3	2.73	3.09	2.79	2.81	3.11	2.73	1.40	2.97	2.97	0.00
NOx-p-CH_3	2.75	3.01	2.84	2.75	3.05	2.68	1.53	3.00	3.00	0.00
NOx-m-Cl	2.81	3.10	2.82	2.87	3.20	2.54	1.47	2.86	3.03	0.00
NOx-o-Cl	2.53	3.19	2.57	3.03	3.19	2.87	1.59	2.83	2.96	0.00
NOx-p-Cl	2.69	3.08	2.89	2.88	3.09	2.65	1.60	2.89	2.94	0.00
NOx-m-NO_2	2.52	3.14	2.64	3.02	3.12	2.82	1.64	2.80	2.98	0.00
NOx-o-NO_2	2.77	3.12	2.73	2.86	3.27	2.59	1.65	2.86	2.90	0.00
NOx-p-NO_2	2.69	3.04	2.88	2.87	3.04	2.67	1.59	2.94	2.97	0.00
NOx-m-OH	2.52	3.25	2.60	3.06	3.15	2.76	1.65	2.87	2.88	0.00
NOx-o-OH	2.81	3.13	2.73	2.84	3.19	2.74	1.57	2.84	2.93	0.00
NOx-p-OH	2.88	2.98	3.05	2.67	2.67	3.18	1.58	2.90	2.93	0.00

Table S7: Electronic populations calculated over bonds and non-bonding regions using the parti-tioning provided by the ELF for the MECI point in all studied systems.

**System**	**C1-N6**	**C1-C2**	**C2-C3**	**C3-C4**	**C4-C5**	**C5-N**	**N-O**	**O1**	**O2**	**N6**
NOx	2.0	2.8	3.1	2.2	3.4	2.0	0.9	2.8	2.8	2.2
NOx-m-CH_3	2.1	3.0	3.0	2.3	3.5	2.0	0.9	2.8	2.8	2.1
NOx-o-CH_3	2.0	2.9	3.0	2.3	3.5	2.0	0.9	2.8	2.8	2.2
NOx-p-CH3	2.0	3.1	2.9	2.3	3.6	2.0	1.0	2.8	2.8	2.1
NOx-m-Cl	2.0	2.8	3.2	2.2	3.4	2.0	1.0	2.8	2.8	2.2
NOx-o-Cl	2.0	2.8	3.1	2.3	3.6	2.0	0.9	2.8	2.8	2.2
NOx-p-Cl	2.0	3.1	3.1	2.4	3.4	2.0	1.0	2.7	2.8	2.1
NOx-m-NO_2	2.0	2.9	3.1	2.3	3.6	2.0	1.0	2.8	2.8	2.2
NOx-o-NO_2	2.0	3.0	3.0	2.2	3.7	2.1	1.0	2.7	2.8	2.1
NOx-p-NO_2	2.0	2.8	3.4	2.2	3.5	2.0	1.0	2.7	2.8	2.1
NOx-m-OH	2.0	3.1	2.9	2.4	3.6	2.0	1.0	2.7	2.8	2.1
NOx-o-OH	2.1	3.0	3.0	2.3	3.6	2.1	0.9	2.8	2.8	2.0
NOx-p-OH	2.0	3.1	3.0	2.4	3.4	2.0	0.9	2.8	2.8	2.1

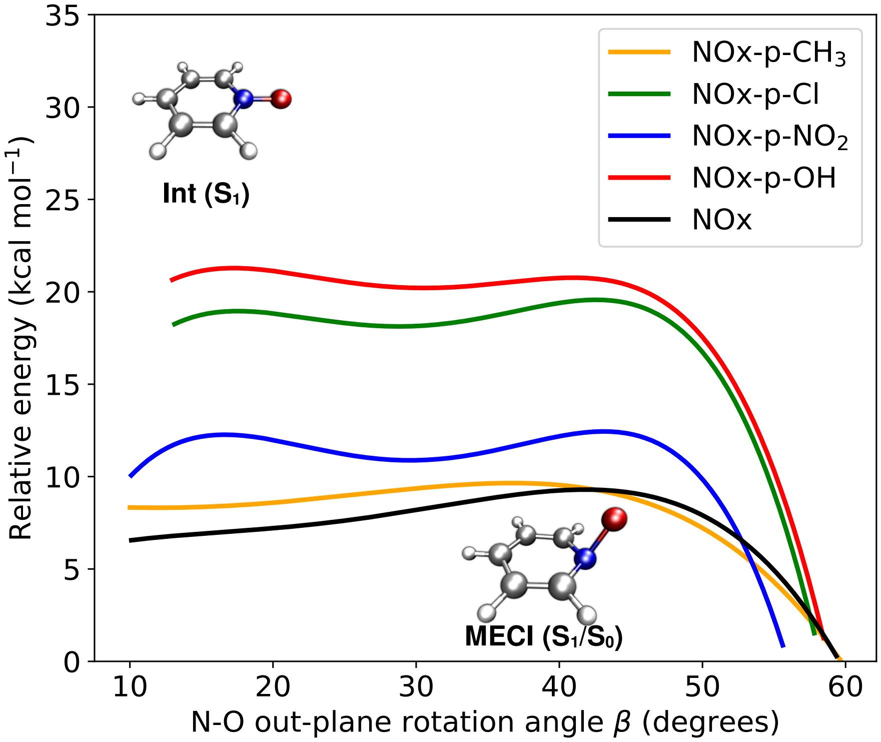

Figure S1: Minimum energy reaction pathway connecting the Int → MECI for the *p*-substituted derivatives. The energies are shown in kcal mol^{-1}, and the reaction coordinates are given in dimensionless units calculated using the IRC method.
